# Phenotypic heterogeneity of neurofibromatosis type 1 in a large international registry

**DOI:** 10.1172/jci.insight.136262

**Published:** 2020-08-20

**Authors:** Mika M. Tabata, Shufeng Li, Pamela Knight, Annette Bakker, Kavita Y. Sarin

**Affiliations:** 1 Department of Dermatology, Stanford University School of Medicine, Redwood City, California, USA.; 2 Children’s Tumor Foundation, New York, New York, USA.

**Keywords:** Dermatology, Neuroscience, Genetic diseases, Genetic variation, Neurological disorders

## Abstract

Neurofibromatosis type 1 (NF1) is a rare genetic disorder, characterized by the development of benign and malignant nerve tumors. Although all individuals with NF1 harbor genetic alterations in the same gene, the clinical manifestations of NF1 are extremely heterogeneous even among individuals who carry identical genetic defects. In order to deepen the understanding of phenotypic manifestations in NF1, we comprehensively characterized the prevalence of 18 phenotypic traits in 2051 adults with NF1 from the Children’s Tumor Foundation’s NF1 registry. We further investigated the coassociation of traits and found positive correlations between spinal neurofibromas and pain, spinal neurofibromas and scoliosis, spinal neurofibromas and optic gliomas, and optic gliomas and sphenoid wing dysplasia. Furthermore, with increasing numbers of cutaneous neurofibromas, the odds ratio of malignant peripheral nerve sheath tumor increased. Phenotypic clustering revealed 6 phenotypic patient cluster subtypes: mild, freckling predominant, neurofibroma predominant, skeletal predominant, late-onset neural severe, and early-onset neural severe, highlighting potential phenotypic subtypes within NF1. Together, our results support potential shared molecular pathogenesis for certain clinical manifestations and illustrate the utility of disease registries for understanding rare diseases.

## Abstract 2

A large clinical phenotypic co-association and cluster analyses is reported for 2051 adult patients with Neurofibromatosis Type 1.

## Introduction

Neurofibromatosis type 1 (NF1) is a rare autosomal dominant genetic disorder affecting 1 in 2500–4000 individuals, characterized by alterations of the neurofibromin (*NF1*) gene located at 17q11.2 (1). Individuals with NF1 may have significant morbidity from the development of nervous system tumors, including neurofibromas, optic gliomas, and malignant nerve sheath tumors. Affected individuals may also experience repeated fractures, cardiac issues, or cognitive disabilities. Among individuals with NF1, the clinical manifestations are highly variable and unpredictable, even among individuals sharing identical genetic *NF1* mutations, suggesting an influence of modifiers outside the *NF1* locus (2). In addition, the prevalence and associations of phenotypic manifestations in NF1 from large adult cohorts are sparse. In addition, no investigations have permitted identification of clinically based subtype patterns, and there is a need to understand how genetic variants both inside and outside the *NF1* locus influence disease subtypes.

To address this knowledge gap, we undertook a comprehensive analysis of NF1 phenotypic trait prevalence and coassociations and explored the presence of disease subtypes, using phenotypic and genetic data from the large NF1 patient population available in the international Children’s Tumor Foundation NF Registry (3). Understanding clinical trait associations and disease subtypes will ultimately pave the way for more informed and personalized disease management.

## Results

### Prevalence of NF1 features.

Our analysis cohort consisted of 2051 patients with a mean age of 42 years (standard deviation of 14 years), and 67% were female (Methods). Table 1 presents prevalence of clinical features, including café au lait macules (98%), cutaneous neurofibromas (cNFs) (91%), scoliosis (45%), plexiform neurofibromas (43%), fractures (36%), spinal neurofibromas (35%), optic gliomas (18%), osteoporosis (14%), malignant peripheral nerve sheath tumor (MPNST) (4%), and sphenoid wing dysplasia (3%) and comparison with previously reported prevalence.

### Associations among phenotypic traits/characteristics.

**Figure 1:**
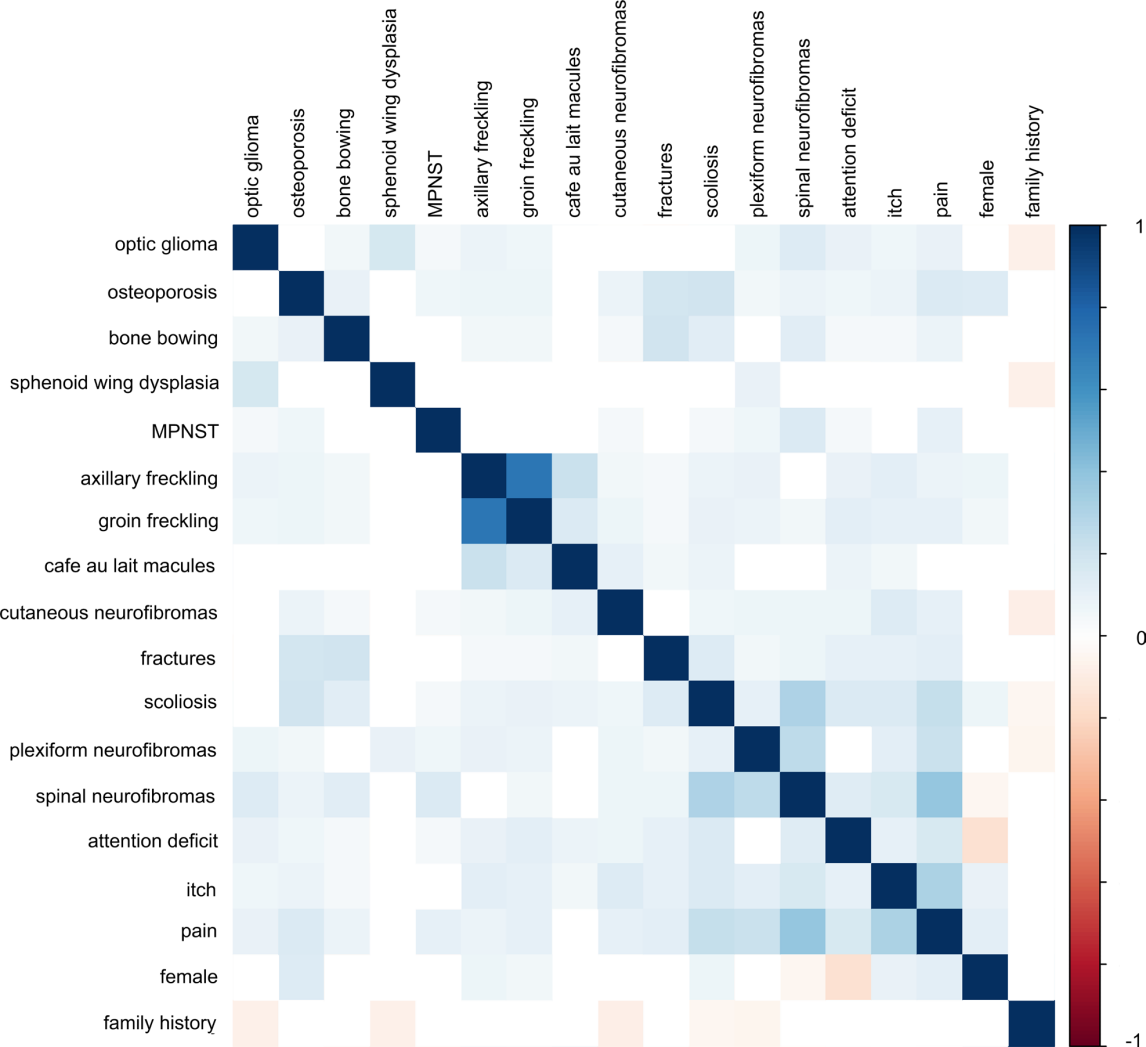
Association of clinical traits by Pearson correlation. An 18 × 18 matrix displays the *r* value by Pearson correlation of each pairwise clinical trait coassociation, using 2051 patient samples. The shade of each cell corresponds to the *r* value of the Pearson correlation between the traits on the corresponding row and column. Only associations significant to *P* < 1.73e4 (after Bonferroni’s correction for multiple comparisons) were plotted. Strong associations include axillary and groin freckling, optic glioma and sphenoid wing dysplasia, spinal neurofibroma and scoliosis, and spinal neurofibroma and pain.

Previous studies have found coassociation of phenotypic traits in NF1, such as plexiform neurofibromas and MPNST, suggesting that multiple phenotypes may share common molecular pathways. In order to understand more broadly how clinical traits associated with one another, we performed Pearson correlations between each pair of traits. Of particular interest, we found strong positive relationships of spinal neurofibromas with pain (*r* = 0.39, *P* < 0.05; OR = 5.25 [CI: 4.02, 6.85], *P* < 0.001), scoliosis (*r* = 0.31, *P* < 0.05; OR = 3.01 [2.42, 3.74], *P* < 0.001), plexiform neurofibromas (*r* = 0.26, *P* < 0.05; OR = 2.74 [2.17, 3.46], *P* < 0.001), and optic gliomas (*r* = 0.15, *P* < 0.05; OR = 2.00 [1.52, 2.64], *P* < 0.001) (Figure 1). Interestingly, optic gliomas were positively associated with sphenoid wing dysplasia (*r* = 0.18, *P* < 0.05; OR = 6.48 [3.57, 11.75], *P* < 0.001) (Figure 1). We did not observe a correlation between optic gliomas and learning difficulties, which was a potential correlation in a prior study (4).

**Figure 2:**
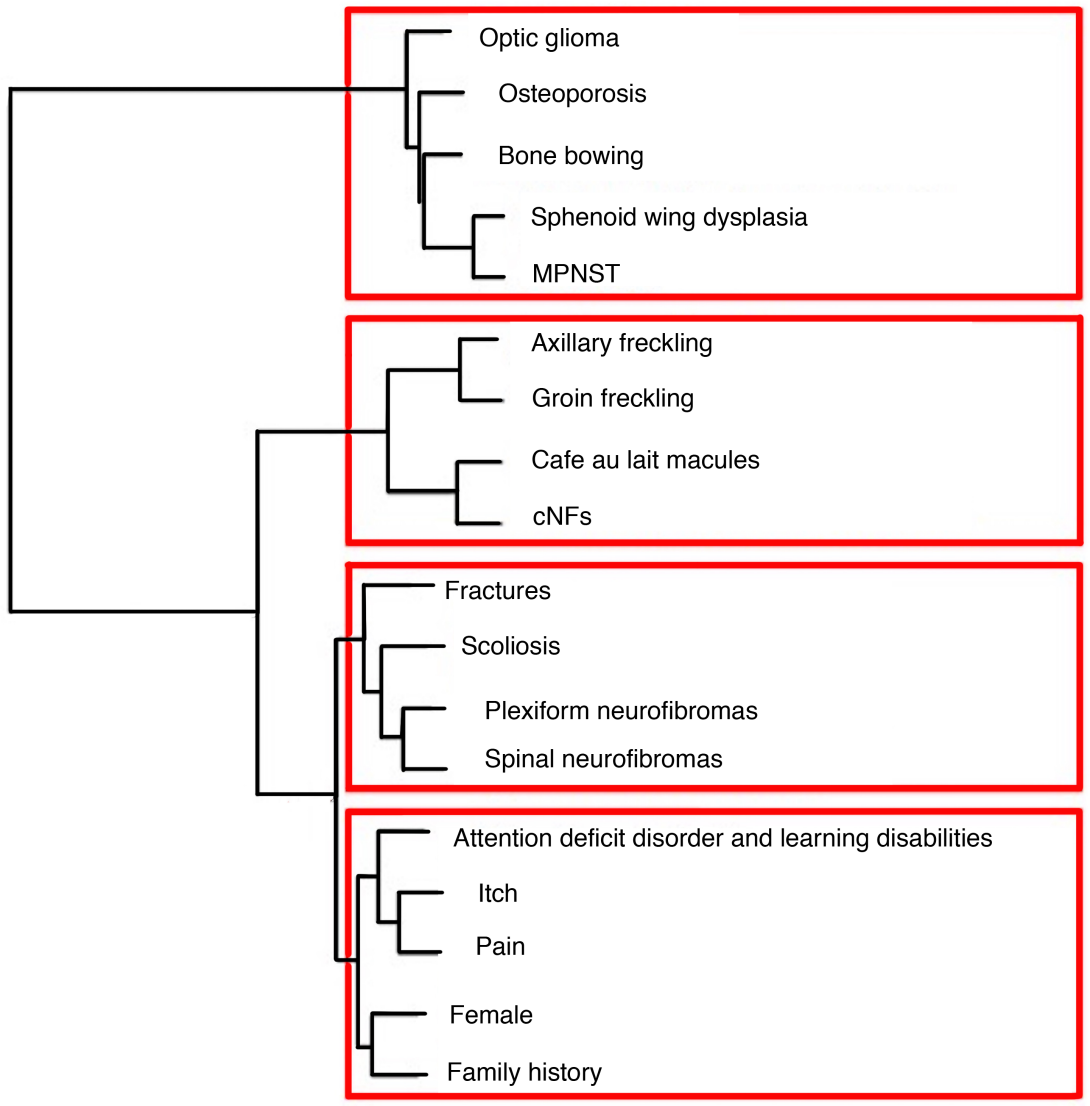
Hierarchical clustering of traits reveals consistency with correlations. Hierarchical clustering with Ward D method shows how traits with the highest degree of similarity across 2051 patients cluster together.

We also sought to identify how phenotypic traits cluster together. We thus performed unsupervised hierarchical clustering of traits, which resulted in 4 major clusters: (a) optic gliomas, osteoporosis, bone bowing, sphenoid wing dysplasia, MPNST; (b) axillary freckling, groin freckling, café au lait macules, cNFs; (c) fractures, scoliosis, plexiform neurofibromas, spinal neurofibromas; (d) attention deficit disorder (ADD), itch, pain, female sex, family history of NF1 (Figure 2). Cutaneous findings clustered and spinal findings clustered, demonstrating that these findings are likely to co-occur.

### Cutaneous findings can inform risk of systemic disease.

Previous reports have shown the absence of cNFs was associated with increased risk of internal malignancy and mortality (5, 6), suggesting that cutaneous findings may provide insight into underlying systemic traits. To explore this further, we performed logistic regression to identify internal traits associated with cutaneous findings. Cutaneous findings were associated with systemic disease after adjustment for all possible confounders. The number of cNFs was significantly associated with itch (OR = 3.74 [2.51, 5.60], *P* < 0.001) and marginally associated with pain (OR = 1.55 [1.00, 2.39], *P* < 0.001), ADD (OR = 1.51 [1.00, 2.27], *P* < 0.001), and plexiform neurofibromas (OR = 1.73 [1.16, 2.59], *P* < 0.001), after adjustment for age and other covariates (Table 2). Additionally, increasing cNFs were associated with MPNST, after adjustment for age and other covariates (cNF number 1 to 10: coefficient = 1.058, OR = 2.88 [0.74, 19.09]; cNF number 11 to 100: coefficient = 1.289, OR = 3.63 [1.01, 23.37]; cNF number > 100: coefficient = 1.46, OR = 4.30 [1.17, 27.93]), although with wide CIs. This association remained when combining those patients with no cNFs and those with 1–10 cNFs as the reference group for comparison and adjusting for age; there was still an increased risk of MPNST with 10–100 cNFs (coefficient = 0.451, OR = 1.568, *P* = 0.191) and with more than 100 cNFs (coefficient = 0.877, OR = 2.404, *P* = 0.015). The number of cNFs showed an inverse association with optic gliomas with increasing numbers of cNFs (Table 2). These results remained unaffected to 3 decimal places after adjustment for the presence or absence of genetic testing confirming NF1.

Skinfold freckling was weakly associated with optic gliomas (OR = 1.69 [1.09, 2.72]), itch (OR = 1.38 [1.02, 1.86]), and plexiform neurofibromas (OR = 1.36 [1.01, 1.82]) (Table 2). Café au lait macules were associated with scoliosis (OR = 3.83 [1.38, 13.68]), ADD (OR = 2.68 [1.27, 5.86]), and family history of NF1 (OR = 2.32 [1.14, 4.72]) (Table 2).

### Phenotypic clustering reveals subtypes of disease.

**Figure 3:**
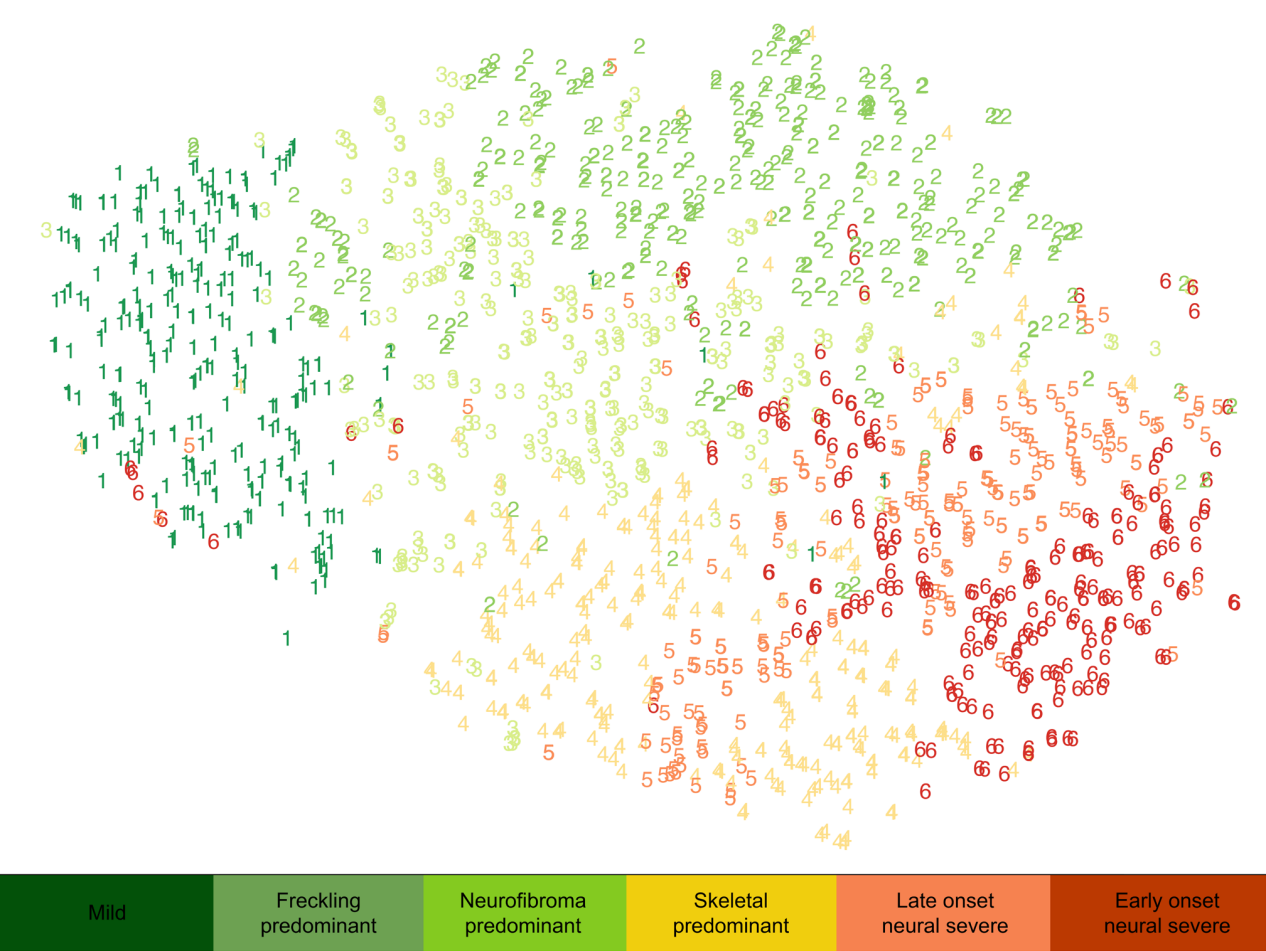
Clustering reveals phenotypic subtypes visualized by t-stochastic neighbor embedding plot. K-means clustering was performed on the principal components of all traits for 2051 patients and resulted in 6 distinct clusters of patients, representing subtypes of NF1. Clusters were visualized using t-stochastic neighbor embedding (t-SNE) method of dimensionality reduction to create a 2-dimensional plot. Clusters were color-coded by severity of subtype, with green being the least severe and red being the most severe, and the separation of colors visually represents the distinctness of clusters.

NF1 is a heterogeneous disease, with individuals displaying varied penetrance of phenotypic traits. We were interested in determining whether patient “subtypes” were present within NF1, defined by common phenotypic manifestations. We thus investigated whether patients form distinct groups based on their phenotypic expression of different clinical traits. K-means clustering of individuals revealed separation of patients into 6 clusters based on clinical characteristics (Figure 3, Supplemental Figure 4, and Supplemental Figure 5; supplemental material available online with this article; https://doi.org/10.1172/jci.insight.136262DS1). Cluster 1, defined here as the “mild” subtype (cluster 1, *n* = 309), is characterized by the mildest overall presentation, manifesting lower prevalence of freckling (3%), cNFs, ADD (54%), and plexiform neurofibromas (32%). Full characteristics are listed in Supplemental Figure 5. Cluster 2, the “freckling-predominant” subtype (cluster 2, *n* = 467), is characterized by overall mild presentation, low prevalence of cNFs and MPNST (1%), but high prevalence of freckling (97%). The “neurofibroma-predominant” subtype (cluster 3, *n* = 409) is characterized by overall mild presentation, with high prevalence of cNFs, itch (88%), and pain (67%) and low prevalence of MPNST (1%). The “skeletal-predominant” subtype (cluster 4, *n* = 344) is characterized by high prevalence of fractures (97%), osteoporosis (33%), and scoliosis (74%). The “late-onset severe neural” subtype (cluster 5, *n* = 250) is characterized by later age of diagnosis and high prevalence of spinal neurofibromas (91%), plexiform neurofibromas (69%), and MPNST (7%). The “early-onset severe neural” subtype (cluster 6, *n* = 272) is the most severe group, diagnosed at early ages and having high prevalence of optic gliomas (58%) and ADD (89%) in addition to spinal neurofibromas (78%), plexiform neurofibromas (78%), and MPNST (7%).

### Mutations in the NF1 gene do not fully explain phenotypic heterogeneity.

Sixty-one individuals with NF1 submitted genetic data to the NF1 registry, and 8 were greater than 18 years of age. From these subjects 43 unique mutations in or involving the *NF1* gene were identified. We were unable to identify any statistically significant associations between the *NF1* mutation and the clinical phenotypes of these patients. In fact 2 pairs of patients had identical phenotypes with different mutations, whereas 1 pair of patients had identical mutations but varied clinical phenotypes (Supplemental Figure 6). This supports prior studies that demonstrated phenotypes are not solely explained by mutations within the *NF1* locus. Interestingly, we did identify an individual in our cohort with *NF1* c.2970_2972delAAT. This mutation is associated with a lack of cNFs or plexiform neurofibromas (7). Accordingly, this individual had a mild phenotype with only freckling, scoliosis, and ADD by 17 years of age. Two patients also harbored *NF1* c.2540T>C (p.Leu847Pro) mutations, which have been associated with freckling, plexiform neurofibromas, and learning disabilities (8). These individuals presented with plexiform neurofibromas and developmental delay consistent with this phenotype (8). Finally, 1 patient harbored a contiguous gene deletion of *NF1* along with neighboring genes. This genetic alteration has been shown to cause a severe NF1 phenotype with increased risk of MPNST, subcutaneous neurofibromas, spinal neurofibromas, and plexiform neurofibromas (9). Accordingly, this subject developed MPNST, plexiform neurofibromas, and spinal neurofibromas, consistent with the severe phenotype in these individuals (9).

## Discussion

In this comprehensive analysis of NF1 clinical heterogeneity in a large adult patient cohort, we have documented the prevalence of 18 phenotypic traits, identified a number of strong coassociations among these traits, and defined 6 likely disease subtypes with what is likely the first clustering analysis of NF1 patients. Together our findings extend understanding of NF1 heterogeneity by identifying certain traits that preferentially present together in NF1, which may enable future risk stratification and thus help inform more precise clinical practice.

Our prevalence findings are largely consistent with those from prior studies (10–22) and validate results from the earlier smaller cohorts. Our cohort has a higher prevalence of scoliosis, possibly because our cohort had an older average age, whereas other studies included pediatric populations, in whom scoliosis was less common. Similarly, the prevalence of MPNST in our study may be slightly lower than in prior studies because of the older age distribution of our cohort, as MPNST has both a young age of onset (median of 28 years) and a tendency for early mortality (5-year survival rate of 39%) (23). Compared with data from the French Clinical Research Program NF1 database published in 2009 with 750 patients, we have a greater prevalence of plexiform neurofibromas (43% vs. 34%), cNFs (91% vs. 62%), and neoplasm (18% optic gliomas vs. 10% any neoplasm), likely due to the older age of our adult-only cohort (24). We also report a greater prevalence of ADD and learning disabilities (70% vs. 48%), and this is likely attributed to difference in definition. Our data are self-reported, and the French Clinical Research Program NF1 database defined learning disability as “referral for remedial education” (24).

Although findings in 1993 in a cohort of 175 individuals did not reveal any associations between cNFs, plexiform neurofibromas, optic gliomas, scoliosis, epilepsy, remedial education, café au lait macules, and freckling, our larger sample size allowed us to detect trait associations (4). We found that optic gliomas associate with sphenoid wing dysplasia and that optic gliomas hierarchically cluster with bony abnormalities. These findings are supported by a prior report showing that nearly half of patients with missense variants in NF1 codons 844–848 had optic gliomas and/or skeletal abnormalities (8). It is possible these phenotypes share common molecular and genetic etiologies. Despite numerous studies of *NF1* gene mutations, allelic heterogeneity alone does not explain phenotypic coassociations. This suggests that phenotypic modifiers occur outside the *NF1* locus (2, 4, 25), and further genetic studies are required to understand these modifiers. Another possibility is that optic gliomas lead to sphenoid wing dysplasia due to mass effect. Additionally, we found optic gliomas positively associate with spinal neurofibromas, suggesting a novel association of optic gliomas not previously reported to our knowledge. Spinal neurofibromas also associate strongly with pain and scoliosis, suggesting that spinal neurofibromas may contribute to scoliosis, although causation cannot be determined with current data. Although we adjusted for confounders in all analyses, we cannot account for other factors that may contribute to trait associations, such as environmental exposures, stochastic events, and epigenetics.

In contrast to the hierarchical clustering of traits in Figure 2, which shows how traits are related to one another, we also performed clustering of individuals by phenotypic traits, which demonstrated how individuals are related to one another. Our patient cluster analysis identified 6 disease subtypes that help inform disease heterogeneity. The mild subtype (cluster 1) is consistent with the mild subtype previously identified in association with 3-nt deletion in exon 17, with lack of neurofibromas and lack of noncardiac comorbidities (7, 26, 27). The freckling-predominant phenotype (cluster 2) is consistent with the c.5425C>T missense variant mild phenotype previously identified with freckling but absence of cutaneous or plexiform neurofibromas, osseous lesions, or optic gliomas (28, 29). The early-onset neural severe subtype (cluster 6) is consistent with the severe phenotype identified in association with missense variants in cysteine-serine-rich domains, characterized by plexiform and spinal neurofibromas, optic gliomas, and an increased risk of malignancy (8). The strong overlap of these described phenotypes associated with genetic alterations highlights the potential impact of genetic variants both in *NF1* and outside loci in modifying NF1 traits and influencing disease subtypes. Since 1993, evidence has suggested that genetic modifiers play a major role in phenotypic variability, when Easton et al. observed high phenotypic similarity in monozygotic twins but not in distant relatives sharing the same NF1 mutation (4). Mice heterozygous for an *NF1*-knockout mutation (*Nf1^+/–^*) do not develop neurofibromas, MPNST, or other hallmark features of NF1, and studies have proposed mismatch repair genes as modifier genes in NF1 tumor development (25). A number of candidate modifiers have been identified, mostly in tumor samples (2). For example, *ANRIL* has been identified as a potential modifier gene in the pathogenesis of plexiform neurofibromas (30). The mutations involving the *NF1* gene in our present study also do not account for the majority of phenotypic presentations (Supplemental Figure 6), providing further evidence that pathway modifications occur outside the NF1 locus. Replication of the clusters identified in our study in an independent cohort should be performed as new registry data become available, and the development of a genetic biobank could be used to identify genetic modifiers of phenotypic traits in NF1. While clusters alone cannot be used to dictate clinical management, taken in conjunction with prior knowledge as described above, they further our understanding of NF1 heterogeneity.

It is important for clinicians to be aware of the association of cutaneous findings with internal disease, especially optic gliomas, plexiform neurofibromas, spinal neurofibromas, and MPNST, which have high morbidity and mortality. Physicians who treat NF1 understand the varying degrees of burden that cNFs can have on patients’ lives, but our findings that cNF associates with ADD, itch, and pain highlights how broadly these cutaneous lesions may influence affected individuals’ quality of life. Consistent with this association, the neurofibroma-predominant subtype also had a high prevalence of itch and pain, which underscores that cNFs can contribute to symptoms and be indicative of disease severity. However, our findings also suggest that lack of cNFs does not preclude learning difficulties. Although the freckling-predominant subtype (cluster 2) had lower prevalences of cNFs and plexiform neurofibromas, 66% of these patients reported having ADD. This observation is consistent both with a patient in our study (Supplemental Figure 6) and with findings of a recent study that demonstrated that individuals with the NF1 c.2970_2972delAAT pathogenic variant had a mild phenotype, lacking plexiform, cutaneous, or subcutaneous neurofibromas but including learning difficulties (26).

Although prior studies reported that lack of cNFs was associated with increased risk for internal malignancy in 208 patients (6) and increased mortality in 378 patients (5), our findings in a much larger cohort show that as the number of cNFs increases in an ordinal fashion, the OR for MPNST increases. This discrepancy can be reconciled by the existence of different subtypes of disease. The neurofibroma-predominant subtype (cluster 3) has a high prevalence of patients with more than 100 cNFs (47%) and low prevalence of MPNST (1%). However, the freckling-predominant subtype (cluster 2) has the lowest prevalence of cNFs among all clusters and a low prevalence of MPNST. It is possible that subtyping disease can lead to different risk stratification algorithms for different groups of patients, and cutaneous findings may need to be interpreted in the context of other findings to be useful predictors of systemic disease.

Our study enjoyed the strengths of a large sample size, which enabled sufficient statistical power to detect important associations, and detailed data on NF1 comorbidities for establishing meaningful disease subtypes. However, it is important to recognize the limitations of survey-based registry studies. Phenotypic data and genetic confirmation were self-reported and not adjudicated by clinical records. The diagnosis of NF1 was confirmed using clinical criteria and genetic testing was not required. Although unlikely, it is possible that a portion of the remaining participants have been misdiagnosed. In particular, participants with no cNFs may have Legius syndrome. However, 1046 participants reported that genetic testing confirmed the diagnosis of NF1. There is also possible selection bias because the participants who completed the survey may not be entirely representative of the entire NF1 community. However, the similar prevalence of NF1 traits seen in our data as compared with previously published, physician-adjudicated data from smaller cohorts suggests that this bias may not be significant. While our unsupervised clusters identified compelling evidence that subtypes of disease exist, these clusters cannot be validated against a “correct solution” and must be examined in the context of the disease. Cluster analysis may also have been influenced by potential bias due to different degrees of medical attention because a patient with MPNST is likely to receive more testing, which would reveal other comorbidities.

Using data from a large international registry, we documented the prevalence of phenotypic traits in adult patients with NF1 and identified coassociations between phenotypic traits that may have useful implications for risk stratification of patients with NF1. Of particular interest, we found that the cNFs and café au lait macules strongly correlate with systemic traits, such as MPNST and scoliosis, indicating that cutaneous findings can inform the risk of systemic disease. We identified phenotypic clusters, suggesting distinct subtypes of disease that may facilitate more personalized treatment, especially with additional genetic and molecular characterizations to further delineate these subgroups. Together, our results support the need for future investigation to uncover possible genetic, molecular, and environmental bases for different presentations of disease and illustrate the utility of large sample sizes in disease registries for understanding rare diseases.

## Methods

### Cohort selection.

Nationwide 3027 participants with NF1 voluntarily registered in the NF1 registry through Children’s Tumor Foundation from June 2012 to June 2018, completing an extensive survey consisting of 48 questions regarding traits of NF1 as part of the registration process (3). Among participants, 96% completed at least 85% of the survey (Supplemental Figure 1), and 1046 had genetic testing that confirmed the diagnosis of NF1. For the current study, registry participants were eligible if they met the diagnosis of NF1 as defined by prior clinical or genetic diagnostic criteria, were older than 18 years at registration and of White race (the only racial group large enough for statistically meaningful analysis), and provided responses to 5 or more of the NF1 key diagnostic criteria; 2051 participants met these study criteria and were included in the analysis (Supplemental Figure 2). For this study group, we performed analysis using data on 18 traits (optic glioma, osteoporosis, bone bowing, sphenoid wing dysplasia, MPNST, axillary freckling, groin freckling, café au lait macules, cNFs, fractures, scoliosis, plexiform neurofibromas, ADD, itch, pain, age, sex, family history).

### Pre-processing and imputation.

For the analytic cohort, the number of cNFs was converted to an ordinal value between 0 and 1, and all other traits were converted to binary values of either 0 or 1. Variables missing from more than 30% of respondents were eliminated from analysis. Missing responses for variables were retained in the analysis, and responses of “not sure” were converted to “no” for MPNST and genetic testing. Missing data comprised 9.6% of the data set, and random forest was used to impute missing responses, yielding a complete data set with 1.8% overall error (31). Missing data are listed by trait in Supplemental Figure 3.

### Statistics.

We evaluated prevalence and associations for the 18 NF1 traits, conducting all analyses in R 3.5.0. First, we defined prevalence as the percentage of included patients who responded yes to having ever received a diagnosis of the trait of interest. Next, to determine trait associations, we calculated Pearson correlation *r* values pairwise for each disease phenotype and plotted an 18 × 18 heatmap of *r* values to visualize associations significant at *P* < 1.73e4, which was the *P* value cutoff after Bonferroni’s correction for multiple hypothesis testing. To further investigate disease associations adjusted for all confounders, including age and sex, we conducted logistic regression; ORs and 95% CIs were calculated from the coefficients of generalized logistic regressions. Confounders were identified by testing individually for variables that changed the OR by 10% or greater. Age and sex were included as covariates in all analyses for potential confounding. Because prior reports suggested associations with the presence of cNFs, we performed separate logistic regressions to identify any internal traits associated with cutaneous findings. We performed unsupervised hierarchical clustering with Euclidean distance and Ward D method for the 18 traits, represented as binary variables. Multiple hypothesis testing was adjusted for with Bonferroni’s correction.

Patient clustering was performed with K-means clustering based on principal components analysis (32). K = 6 was chosen as the optimal number of clusters because it represented the inflection point by elbow plot. Additionally, stability analysis of clusters using the clValid package in R 3.5.0 revealed that the average distance and figure of merit were optimized when K = 6. Dimensionality reduction and data visualization of the resulting clusters were performed with t-SNE plots, color-coded by cluster. Clusters were named according to differentiating characteristics.

### Study approval.

Study approval and informed consent were not required by the Institutional Review Board at the authors’ institution because the data were obtained previously by the Children’s Tumor Foundation, at which time consent was obtained to use the data for research.

## Author contributions

MMT performed data analysis, manuscript writing, and manuscript editing. SL performed statistical supervision and assisted in manuscript revision. PK performed a major role in the data acquisition and assisted in manuscript revision. AB performed a major role in the data acquisition and assisted in manuscript revision. KYS designed and conceptualized the study, supervised data analysis, and revised the manuscript for intellectual content.

## Supplementary Material

Supplemental data

## Figures and Tables

**Table 2:**
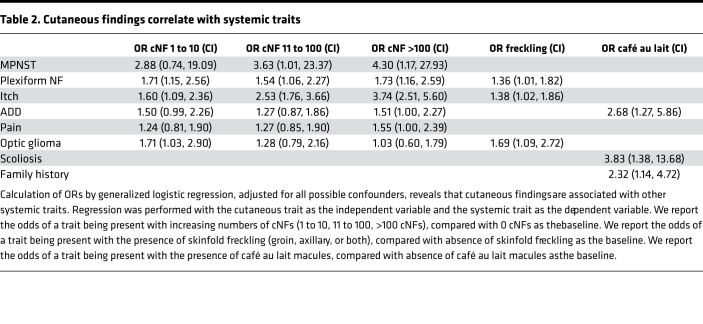
Cutaneous findings correlate with systemic traits

**Table 1:**
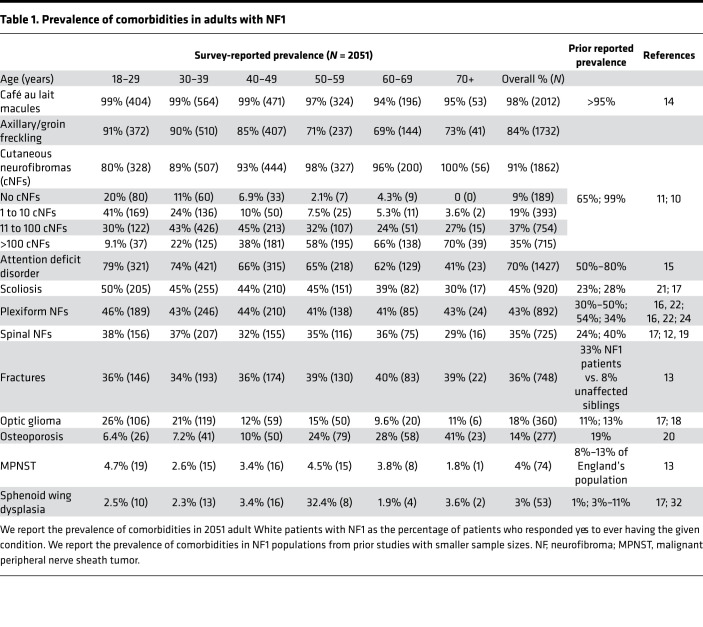
Prevalence of comorbidities in adults with NF1

## Version 1 08/20/2020

Electronic publication
