# Supplementary material for: Phenotypic heterogeneity of neurofibromatosis type 1 in a large international registry
Source: JCI Insight. 2020 Aug 20;5(16):e136262. doi: 10.1172/jci.insight.136262 (PMC7455126; doi:10.1172/jci.insight.136262)
Supplement: Supplemental data [file jciinsight-5-136262-s078.pdf]

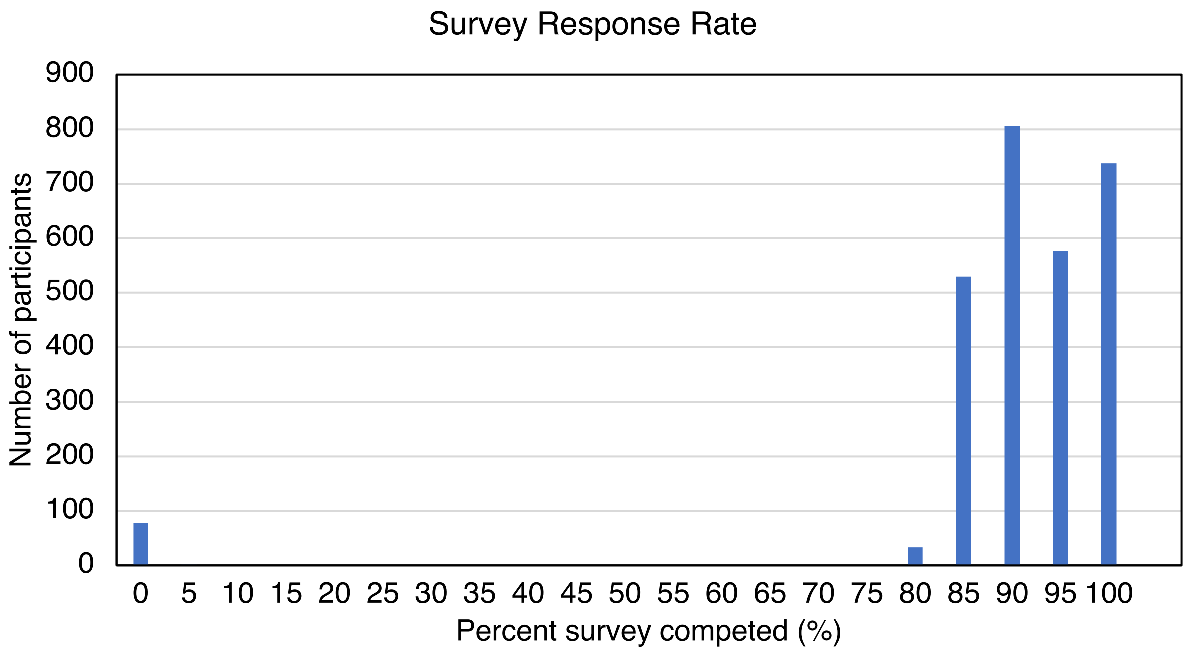

Supplementary Figure 1. Survey response rate. Histogram depicting the response rate of the participants, calculated as the percent of the survey completed out of 48 total questions.

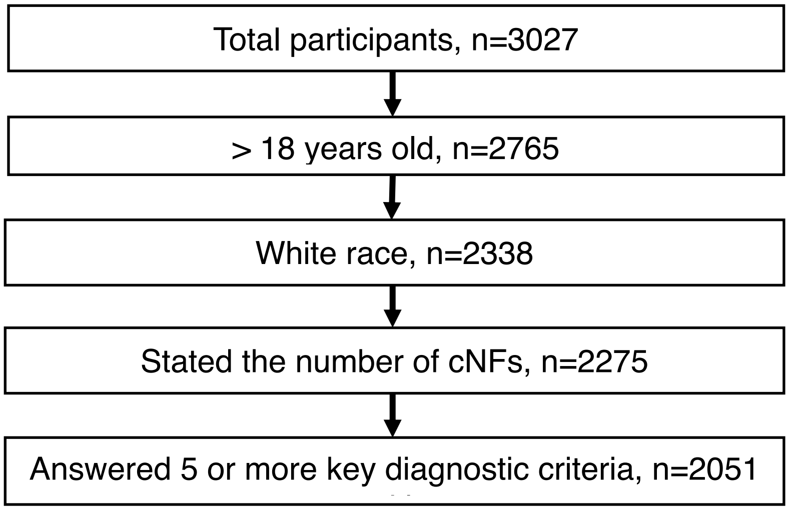

Supplementary Figure 2. Schematic of cohort identification**.** 3027 surveys from unique individuals with NF1 were provided by the Children's Tumor Foundation. Of these, 2051 were white individuals greater than 18 years of age who responded to 5 or more NF1 diagnostic criteria and stated their number of cNFs. Key diagnostic criteria for which at least 5 responses were required included café au lait macules, freckling, neurofibromas of any type, optic gliomas, osseous lesions, and family history of first degree relative. cNF= cutaneous neurofibroma

Characteristic	Number of responses (N, %)
Age of diagnosis	1937 (94.4%)
Axillary freckling	1844 (89.9%)
Groin freckling	1705 (83.1%)
Café au lait macules	2040 (99.5%)
Cutaneous neurofibromas	2051 (100%)
Plexiform neurofibromas	1420 (69.2%)
Optic gliomas	1691 (82.4%)
Sphenoid wing dysplasia	1781 (86.8%)
Fractures	1950 (95.1%)
Osteoporosis	1704 (83.1%)
Scoliosis	1829 (89.2%)
Bone bowing	1734 (84.5%)
Spinal neurofibromas	1631 (79.5%)
Itch	1838 (89.6%)
Pain	2051 (100%)
Attention deficit and learning disabilities	1636 (79.8%)
MPNST	2051 (100%)
Family history	1895 (92.4%)

Supplementary Figure 3**.** Survey data responses. Number of responses for each trait in the 2051 patient cohort. A response was considered missing if there was either a response of “not sure” or no response by the participant. MPNST = malignant peripheral nerve sheath tumor.

1: Mild (n=309)	3% freckling (84%)<br>15% no cutaneous NFs (9%)<br>54% attention deficit (70%)
2: Freckling predominant (n=467)	97% freckling (84%)<br>17% >100 cutaneous NFs (35%)<br>2% osteoporosis (14%)<br>1% MPNST (4%)
3: Neurofibroma predominant (n=409)	78% >10yrs at diagnosis (37%)<br>47% >100 cutaneous NFs (35%)<br>1% MPNST (4%)<br>88% itch (68%)<br>67% pain (61%)
4: Skeletal predominant (n=344)	97% fractures (36%)<br>33% osteoporosis (14%)<br>74% scoliosis (45%)<br>93% itch (68%)<br>85% attention deficit (70%)
5: Late-onset neural severe (n=250)	91% spinal NF (35%)<br>69% plexiform NF (43%)<br>7% MPNST (4%)<br>77% scoliosis (45%)<br>90% family (62%)
6: Early-onset neural severe (n=272)	78% spinal NF (35%)<br>78% plexiform NF (43%)<br>7% MPNST (4%)<br>6% >10 yrs at diagnosis (37%)<br>58% optic glioma (18%)<br>8% sphenoid wing dysplasia (3%)<br>89% attention deficit (70%)

Supplementary Figure 4. Summary characteristics of six subtypes of NF1, represented as the subtype percentage compared to the full cohort average percentage. The right column displays the percentage of patients in the subtype with the trait of interest followed by the percentage of the full cohort that has the trait of interest with the format: subtype% (full cohort%).

NF = neurofibroma. MPNST = malignant peripheral nerve sheath tumor.

	1 – Mild	2 – Freckling predominant	3 –NF predominant	4- Skeletal predominant	5 – Late onset neural severe	6 – Early onset neural severe	Total	
n	309	467	409	344	250	272	2051	
age	48.59	37.66	47.11	41.56	43.2	35.81	42.27 (Avg%)	SD
female	178 (58%)	286 (61%)	313 (77%)	296 (86%)	150 (60%)	150 (55%)	1373 (67%)	12%
diagnosis	167 (54%)	79 (17%)	319 (78%)	48 (14%)	126 (50%)	17 (6%)	756 (37%)	28%
axillary	3 (1%)	452 (97%)	399 (98%)	330 (96%)	241 (96%)	260 (96%)	1685 (82%)	39%
groin	8 (3%)	425 (91%)	358 (88%)	323 (94%)	237 (95%)	250 (92%)	1601 (78%)	37%
CAL	278 (90%)	467 (100%)	403 (99%)	344 (100%)	249 (100%)	271 (100%)	2012 (98%)	4%
cNF								
0	45 (15%)	75 (16%)	19 (5%)	17 (5%)	17 (7%)	16 (6%)	189 (9%)	5%
1-10	45 (15%)	116 (25%)	54 (13%)	66 (19%)	50 (20%)	62 (23%)	393 (19%)	5%
11-100	97 (31%)	196 (42%)	143 (35%)	125 (36%)	94 (38%)	99 (36%)	754 (37%)	3%
>100	122 (39%)	80 (17%)	193 (47%)	136 (40%)	89 (36%)	95 (35%)	715 (35%)	10%
PNF	98 (32%)	128 (27%)	111 (27%)	170 (49%)	172 (69%)	213 (78%)	892 (43%)	22%
optic	23 (7%)	74 (16%)	42 (10%)	57 (17%)	7 (3%)	157 (58%)	360 (18%)	20%
sphenoid	6 (2%)	5 (1%)	11 (3%)	8 (2%)	2 (1%)	21 (8%)	53 (3%)	3%
fractures	98 (32%)	114 (24%)	98 (24%)	334 (97%)	63 (25%)	41 (15%)	748 (36%)	30%
osteo	36 (12%)	8 (2%)	55 (13%)	113 (33%)	40 (16%)	25 (9%)	277 (14%)	10%
scoliosis	92 (30%)	142 (30%)	54 (13%)	256 (74%)	192 (77%)	184 (68%)	920 (45%)	27%
bowing	22 (7%)	51 (11%)	11 (3%)	115 (33%)	16 (6%)	50 (18%)	265 (13%)	11%
spine	88 (28%)	20 (4%)	18 (4%)	159 (46%)	228 (91%)	212 (78%)	725 (35%)	37%
itch	183 (59%)	131 (28%)	361 (88%)	320 (93%)	188 (75%)	212 (78%)	1395 (68%)	24%
pain	156 (50%)	50 (11%)	273 (67%)	306 (89%)	231 (92%)	245 (90%)	1261 (61%)	32%
ADD	168 (54%)	307 (66%)	248 (61%)	291 (85%)	171 (68%)	241 (89%)	1426 (70%)	13%
MPNST	13 (4%)	7 (1%)	4 (1%)	13 (4%)	17 (7%)	20 (7%)	74 (4%)	3%
family	187 (61%)	273 (58%)	295 (72%)	241 (70%)	226 (90%)	45 (17%)	1267 (62%)	25%

Supplementary Figure 5. Complete characteristics of six subtypes of NF1. Table of all of the characteristics of each subtype. The left-hand column for each subtype represents the number in the subtype with the trait of interest, and the right-hand columns represents the percentage of patients in the subtype with the trait of interest. The three rightmost columns represent the number of patients, percentage, and standard deviation for each trait out of the full cohort. NF= neurofibroma. CAL = café au lait macules. cNF = cutaneous neurofibroma. PNF = plexiform neurofibroma. Optic = optic glioma. Sphenoid = sphenoid wing dysplasia. Osteo = osteoporosis. Bowing = bowing of bones. ADD = attention deficit disorder and learning disabilities. MPNST = malignant peripheral nerve sheath tumor.

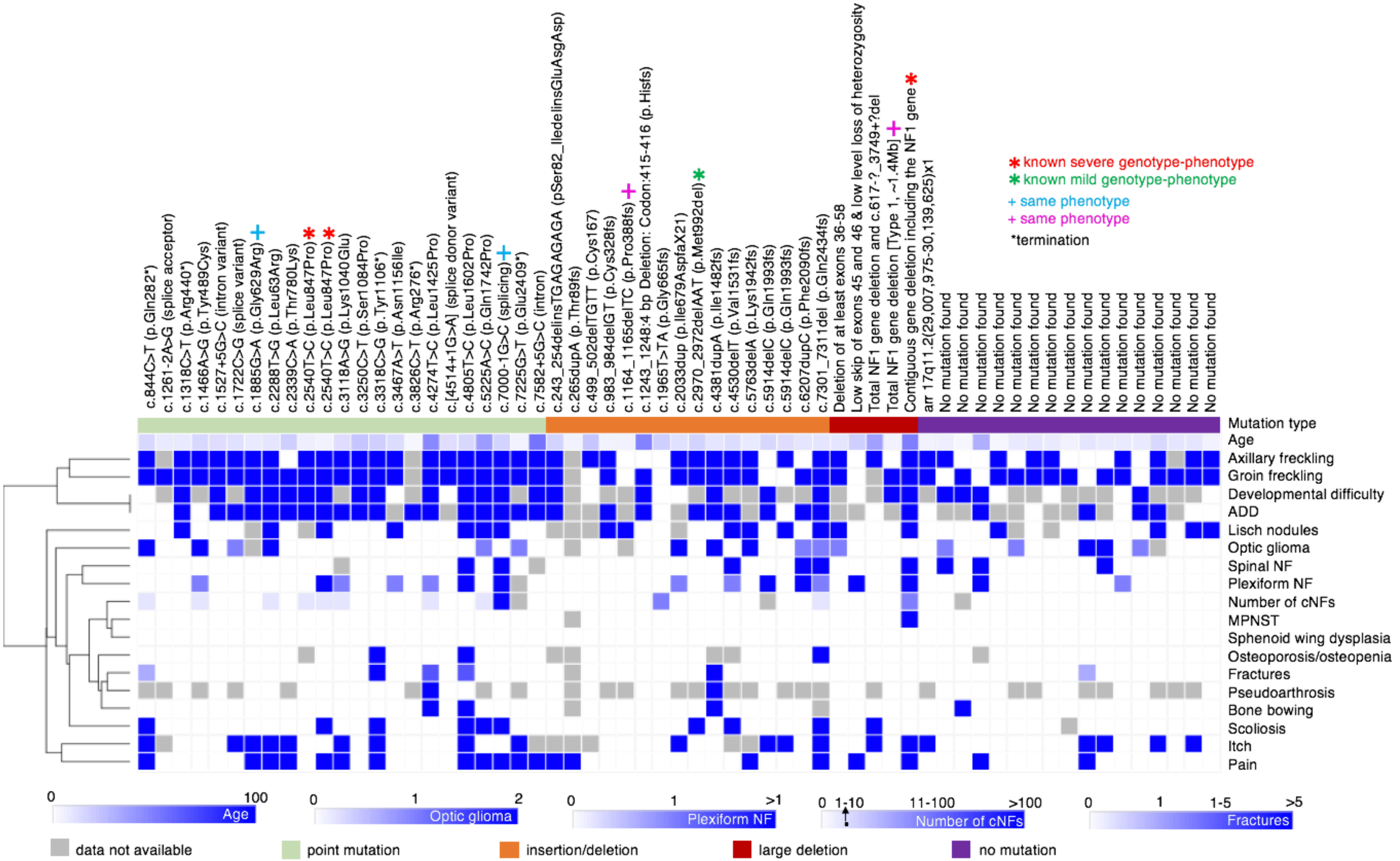

Supplementary Figure 6. Genetic and phenotypic data from 61 individuals with NF1. Rows (phenotypic traits) have been hierarchically clustered. Each column represents a unique patient, labelled with the mutation involving the *NF1* gene. Known phenotypic-genotypic presentations are indicated(*). The same phenotypic presentations with different mutations are indicated(+).
